# SIRPα Suppresses Response to Therapeutic Antibodies by Nurse Like Cells From Chronic Lymphocytic Leukemia Patients

**DOI:** 10.3389/fimmu.2020.610523

**Published:** 2021-01-21

**Authors:** Yu-Chen Enya Chen, Melinda Burgess, Sally Mapp, Peter Mollee, Devinder Gill, Antje Blumenthal, Nicholas A. Saunders

**Affiliations:** ^1^ Diamantina Institute, University of Queensland, Woolloongabba, QLD, Australia; ^2^ Cancer Services Unit, Department of Haematology, Princess Alexandra Hospital, Woolloongabba, QLD, Australia; ^3^ Translational Research Institute, University of Queensland School of Medicine, Woolloongabba, QLD, Australia

**Keywords:** nurse-like-cells, chronic lymphocytic leukemia, macrophages, antibody dependent phagocytosis, antibody resistance 2, macrophage

## Abstract

Targeted antibody therapies improve outcomes for chronic lymphocytic leukemia (CLL) patients. However, resistance often develops. We have previously shown that resistance to therapeutic antibodies, by monocyte derived macrophages (referred to as nurse like cells, NLCs), from CLL patients is characterized by suppression of antibody dependent phagocytosis (ADP). The mechanism(s) contributing to the muted ADP responses remain unresolved. In this regard, an innate immune checkpoint was recently described that uses the CD47:SIRPα axis to suppress phagocytic responses by macrophages. In this study we examine whether the SIRPα axis regulates ADP responses to the anti-CD20 antibody, obinutuzumab, by NLCs. Using siRNA depletion strategies we show that SIRPα is a suppressor of ADP responses. Moreover, we show that this innate immune checkpoint contributes to the resistance phenotype in NLCs derived from CLL patients. Finally, we show that SIRPα suppression is mediated *via* the phosphatase, Shp1, which in turn suppresses SYK-dependent activation of ADP. Thus, we identify a druggable pathway that could be exploited to enhance sensitivity to existing therapeutic antibodies used in CLL. This is the first study to show that activation of the CD47:SIRPα innate immune checkpoint contributes to ADP resistance in NLCs from CLL patients.

## Introduction

Macrophages are a cellular effector of antibody-dependent phagocytosis (ADP) (1–4). Indeed, reports suggest that macrophages may be the major immune effector to clear tumor cells in response to therapeutic antibodies such as obinutuzumab (3, 5–7). Therapeutic antibodies targeting CD20, such as obinutuzumab and rituximab, are used in first- and second-line treatments for CLL (8–12). Although of proven clinical value in CLL treatment, acquired resistance to antibody therapies remains a serious issue and contributes to relapse and treatment failure (13).

Culture of peripheral blood monocytes from chronic lymphocytic leukemia (CLL) patients gives rise to macrophage-like cells referred to as nurse like cells (NLCs). NLCs display profound ADP activity against CLL cells incubated with obinutuzumab or rituximab (4, 14). In this regard, NLCs provide a clinically relevant *ex vivo* model of ADP responses and mechanisms of resistance in CLL (1, 2, 4, 14, 15). For example, NLCs derived from patients with stable/early disease mount a robust ADP response in which 30–60% of the NLCs participate in ADP against obinutuzumab or rituximab-opsonised CLL cells (1, 14). We refer to these NLCs as “sensitive” (1, 14). In contrast, NLCs derived from patients with progressive/relapsed/refractory disease requiring treatment display a muted ADP response to obinutuzumab or rituximab (1, 14). Typically, less than 10% of NLCs participate in ADP responses to antibody-opsonized CLL cells (1, 14). We refer to these NLCs as “resistant.” This resistance to therapeutic antibodies is reversible and actionable (1, 14). For example, the use of FcγRIIB blocking antibodies, HDAC7-selective inhibitors, and SHIP1 inhibitors have all been shown to enhance ADP responses in phenotypically resistant NLCs (1, 16, 17).

ADP responses are initiated when opsonized targets bind an Fcγ Receptor (FcγR) complex on immune effectors such as NLCs (15). FcγRs are a family of receptors comprising activating FcγRs (e.g. FcγR1,2A,3 and 4 in humans) and an inhibitory FcγR2B (15). Once ligated, in the context of CLL, FcγR induce ADP *via* activation of either a SYK/BTK dependent signaling pathway or a PI3K/p110δ signaling pathway (1, 2). The SYK/BTK pathway is muted in phenotypically resistant NLCs whereas the PI3K/p110δ/AKT pathway is unaltered between phenotypically sensitive or resistant NLCs (2). The SYK/BTK mediated resistance can be reversed with Ship1 inhibitors (1). We recently discovered that histone deacetylase 7 (HDAC7) directly suppresses BTK activation in phenotypically resistant NLCs which can be reversed by treatment with HDAC7-selective inhibitors (17). Thus, ADP resistance is actionable and appears to be mediated exclusively by SYK and BTK signaling. Moreover, it is able to be modified with clinically available agents.

Recent evidence suggests that phagocytosis of opsonized or non-opsonized targets occur in the context of an immune synapse-like structure involving the clustering of activating and inhibitory immune receptors (18). Within the FcγR family, there are activating and inhibitory FcγRs which cluster prior to phagocytosis (19–22). Moreover, external to the FcγRs are coregulatory receptors such as SIRPα which are expressed on macrophages and activated by ligation with ligands such as CD47 expressed on a target cell (18, 23). There is growing evidence that the CD47/SIRPα axis may inhibit phagocytosis of tumor cells (7, 23–25). A typical scenario would see CD47 (“don’t eat me” signal) expressed on a cancer cell bind to SIRPα on an immune effector (e.g. macrophage) causing an inhibitory signal in the effector cell which dampens phagocytosis (7, 23). Thus, the CD47:SIRPα axis is referred to as an innate immune checkpoint (23) and is being targeted in a number of clinical trials in various cancer types including AML, breast cancer, CLL, lymphoma and head neck skin cancer (26, 27). Early data emerging from these trials suggests that blood cancers are more sensitive than solid tumors to anti-CD47:SIRPα therapies (15). However, it has also emerged that targeting ubiquitously expressed CD47 has the potential for inducing unwanted side effects such as anaemia (23). Since SIRPα is selectively expressed on myeloid cells (28) targeting this component of the innate immune checkpoint may offer an opportunity to produce good clinical efficacy with fewer side effects. Indeed, therapeutic antibodies have already been developed to target SIRPα (23, 25). Understanding downstream events controlling SIRPα signaling offers the potential to identify new actionable targets that could be used to antagonize the CD47;SIRPα immune checkpoint and improve the specificity and extent of responses to existing therapeutic antibodies.

In this study we explore the possibility that the CD47:SIRPα immune checkpoint may be a negative regulator of ADP responses in NLCs derived from CLL patients. We report, for the first time, that the CD47:SIRPα axis negatively regulates ADP responses in NLCs from CLL patients. We also show that SIRPα-mediated suppression of ADP responses is mediated *via* Shp1/SYK dependent signaling. Thus, we identify new components of the innate immune checkpoint, in NLCs from CLL patients, that could be targeted to enhance responses to therapeutic antibodies in current use in CLL patients.

## Materials and Methods

### CLL Samples and Cultures

Peripheral blood was collected from CLL patients following informed consent according to protocols approved by the Princess Alexandra Hospital (PAH) Human Research Ethics Committee. Diagnosis of CLL was made according to iwCLL criteria (29). Peripheral blood mononuclear cells (PBMCs) were isolated using density gradient centrifugation and cultured as described previously (1). Briefly, CLL PBMCs were cultured at high density (5 × 10^6^ cells/ml) in RPMI-1640 medium, supplemented with 10% heat-inactivated fetal bovine serum (FBS), 100 U/ml penicillin G, 100 ug/ml streptomycin, and 2.92 mg/ml glutamine (all from Thermo Fisher Scientific). Cells were cultured for 7 days to allow maturation of monocytes into NLCs as described previously (1). Cultures were incubated with obinutuzumab (10 µg/ml; GA101; Roche) and/or, anti-human CD47 functional grade antibody (2.5 µg/ml; clone B6H12 from Thermo Fisher Scientific as indicated).

### Reagents

Syk inhibitor (R406; 3 µM) and PI3Kδ inhibitor (Idelalisib CAL-101; 3 µM) were from Selleckchem. SHP-1 inhibitor (TPI-1; 3 µM) and SHP-2 inhibitor (SHP099; 10 µM) were from MedChem Express. Fc-block reagent was purchased from Miltenyi Biotech.

### Antibody-Dependent Phagocytosis Assay

FcγR mediated phagocytosis was examined on CLL PBMC cultures in Nunc Lab-TekII chamber slides (Thermo Fisher Scientific). Following 7 days in culture, cultures were treated with indicated inhibitors or CD47 blockade before the addition of obinutuzumab for an additional 2 h. Non-adherent cells were removed by gentle agitation and washing before being fixed in 4% paraformaldehyde for 10 min. Slides were then stained with May-Grunwald-Giemsa as described previously (1). Slides were analyzed by light microscopy using a DS Fil color camera fitted to a Nikon brightfield microscope (Nikon). Phagocytosis was estimated by counting the number of NLCs that contained phagocytosed CLL cells. A minimum of 200 NLCs per sample were counted and all quantification was performed using ImageJ software.

### Flow Cytometry

To determine the expression of CD47 on CLL cells, PBMCs from CLL patients were stained with APC-CD19 (Clone HIB19), FITC-CD5 (Clone UCHT2), and PE-CD47 (Clone C2CC6; all from Biolegend). CLL cells were gated on CD19+/CD5+ and the percentage of CD47 positivity determined. All samples were analyzed on a BD FACSCanto flow cytometer (BD Biosciences) and data were analyzed using BD FACSDiVa software (BD Biosciences).

### Immunofluorescent Staining

Immunofluorescent staining was performed as described previously (1). CLL PBMCs were cultured for 7 days in 8-well Nunc Lab-Tek II Chamber Slides, and where indicated, were treated with 10 µg/ml obinutuzumab for 30 min. CLL cells were removed by gentle agitation and washing before being fixed in 4% paraformaldehyde. Primary antibodies include mouse monoclonal anti-human CD47 (Clone B6H12.2; Thermo Fisher Scientific) and rabbit monoclonal anti-human SIRPα (Clone EPR16264; Abcam). Secondary antibodies include Alexa Fluor^®^ 555 donkey-anti-rabbit and Alexa Fluor^®^ 488 goat-anti-mouse (both from Thermo Fischer Scientific). Sections were counterstained with DAPI for nuclear staining before being mounted with ProLong Gold (Cell Signaling Technology). Immunofluorescent signals were viewed through an Olympus FV1200 Laser Scanning Confocal Microscope system (Olympus) and analyzed using Olympus FV-10-ASW imaging software. Where indicated, z-stack images were analyzed using ImageJ software.

### Western Blot

Immunoblotting has been described previously (1). Primary antibodies used include: SIRPα (1:1,000; #13379; Cell Signaling), phospho-Shp1 (1:1,000; Tyr564; #8849; Cell Signaling), total Shp1 (1:1,000; #3759; Cell Signaling), phospho-Shp2 (1:1,000; Tyr580; #3703; Cell Signaling), total Shp2 (1:1,000; #3397; Cell Signaling), phospho-Lck (1:1,000; Y394; #ab201567; Abcam), total Lck (1:1,000; #2752; Cell Signaling), phospho-SYK (1:1,000; Tyr525/526; #2710; Cell Signaling), total SYK (1:1,000; #13198; Cell Signaling), phospho-AKT (1:1,000; Ser473, #5082; Cell Signaling), total AKT (1:1,000; #9272; Cell Signaling), GAPDH (1:5,000; #5174; Cell Signaling), and β-actin (1:5,000; sc47778; Santa Cruz Biotechnology). Horseradish peroxidase-conjugated goat anti-rabbit (1:5,000; Cell Signaling) and goat anti-mouse secondary antibodies (1:5,000; Sigma-Aldrich) were used for detection. SignalFire™ Plus ECL reagent (#12630S, Cell Signaling) was used for protein expression detection as per the manufactures protocol. Fusion SL (Analis Instruments) was used as per manufacturer instructions and chemiluminescent captured using Fusion SL (Analis Instruments). Where protein expression has been quantitated, results represent relative protein levels normalized to corresponding total protein levels or β-actin or GAPDH using Image J software.

### siRNA Knockdown

Predesigned stealth small interfering RNAs (siRNAs) were used to silence protein expression of SIRPα (27mer Human siRNA duplexes; SR315411; OriGene) and Shp1 (27mer Human siRNA duplexes; SR321517; OriGene). Non-targeting scrambled siRNA (Trilencer-27 Universal Scrambled Negative Control siRNA Duplex; SR30004; OriGene) was used as a control. On day 7 of culture, non-adherent cells were removed by gentle agitation before NLCs were transfected for 4 h with 50 nM siRNA using Lipofectamine RNAiMAX (LMRNA015; Thermo Fisher Scientific) according to the manufacturers’ instructions. CLL cells were co-cultured with NLCs following transfection protocol. Cells were harvested 48 h post transfection for western blotting or used in functional ADP assays.

### Statistical Analysis

A minimum of triplicate determinations from five biological replicates per treatment group was used, unless otherwise indicated. Statistical differences were calculated using a Mann Whitney U Test or one-way ANOVA followed by Dunnett’s test to account for P values <0.05 using GraphPad Prism 8.4.1.

## Results

### SIRPα and CD47 Expression Are Similar in Cultures of PBMCs From Phenotypically Sensitive and Resistant CLL Patients

**Figure 1:**
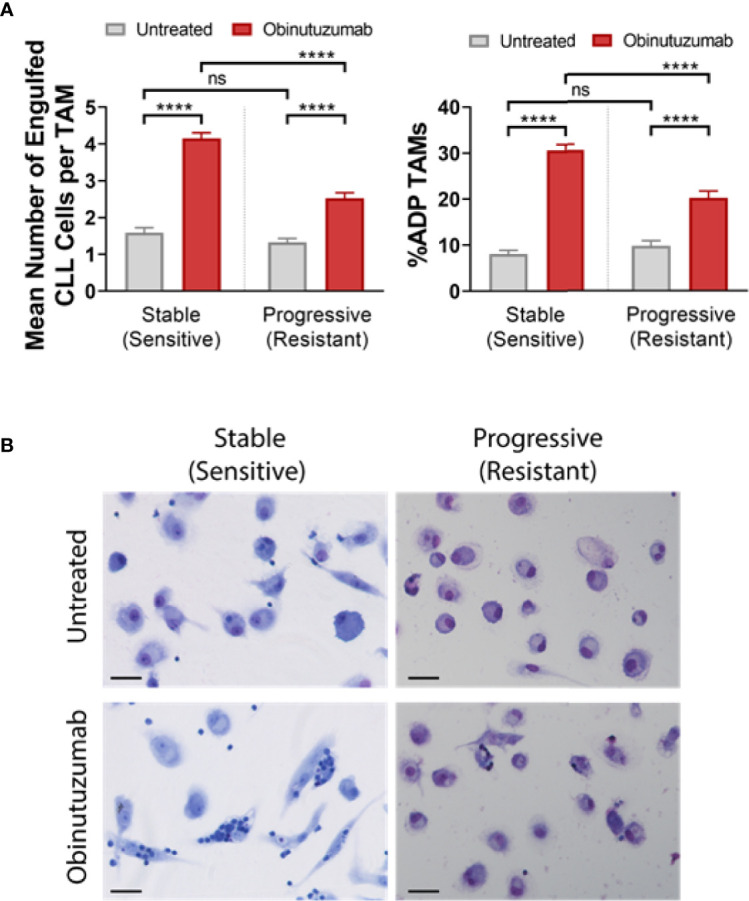
ADP response of stable and progressive CLL derived NLCs. PBMC cultures from stable and progressive disease CLL patients were cultured for 7 days to generate NLCs before stimulated with or without obinutuzumab (10 µg/ml) for 2 h. NLCs were stained with May-Grunwald-Giemsa stain and phagocytosis was quantitated as described in *Materials and Methods*. **(A)** Quantified data presented as the percent NLCs engulfing CLL cells as a fraction of the total pool of NLCs in the culture or the number of engulfed cells per phagocytosing NLC. Data presented as mean value ± SEM from five patients in each group and analyzed by a Mann Whitney U Test. ****p < 0.0001. **(B)** Representative images of May-Grunwald-Giemsa stained stable and active CLL patients’ PBMCs derived NLCs after obinutuzumab treatment. Scale bar = 25μm. ns, not significant.

**Figure 2:**
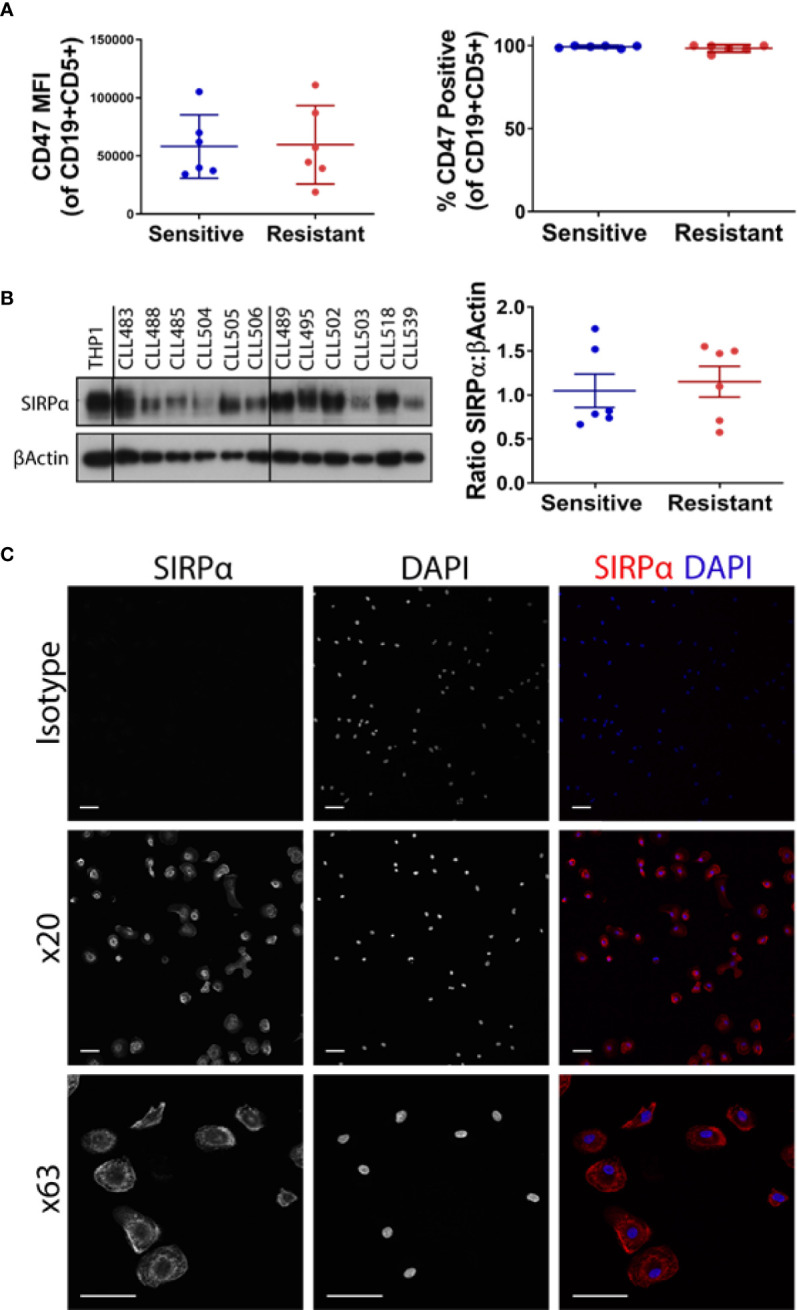
CD47 and SIRPα expression on CLL and NLCs respectively. PBMCs from CLL patients were cultured for 7 days. **(A)** CLL cells were analyzed for CD47 expression by flow cytometry. CLL cells were gated on CD19 and CD5 positivity and data presented as mean fluorescent intensity (MFI) or percentage of CLL cells expressing CD47. CLL cultures have been classified as phenotypically sensitive or antibody resistant as described in *Introduction*. Each point represents an individual patient. The mean value ± SEM is presented. **(B)** NLCs generated on day 7 of CLL PBMC culture were harvested for protein expression of SIRPα by western blot. Western blots are shown for six sensitive and six resistant NLCs, THP-1 is shown as a positive control. Quantified data is shown graphically (right panel). The mean value ± SEM is presented. **(C)** Immunofluorescent staining of SIRPα on NLCs. Cells staining positive for SIRPα appear red and nuclear staining appears blue (DAPI). Representative images from a single patient are shown; ×20 and ×63 magnification. Scale bar = 50 and 100 μm.

We have previously shown that NLCs derived from patients with early/stable disease can be typically classified as phenotypically sensitive in *ex vivo* ADP assays (1). Conversely, NLCs derived from patients with CLL requiring treatment can be typically classified as phenotypically resistant in *ex vivo* ADP assays (1). We now confirm that NLCs become phenotypically “resistant” to ADP during CLL disease progression from stable to progressive disease (1) (**Figures 1A, B**). To confirm our ADP assay only measures phagocytosis we provide a z stack series of IF images with phenotypically sensitive or resistant NLCs showing it discriminates phagocytosis from cell surface binding (**Supplementary Figures 1A, B**). In addition, we confirm that ADP responses are mediated *via* both a SYK/BTK-dependent pathway and a PI3K/p110δ-dependent pathway as characterized by sensitivity to R406 and idelalisib respectively (1, 2) (**Supplementary Figures 2A, B**). To examine whether the CD47:SIRPα immune checkpoint is a negative regulator of ADP in NLCs from CLL patients we initially quantitated leukemic cell surface expression of CD47 by flow cytometry (**Figure 2A**) and NLC SIRPα expression by western blot (**Figure 2B**). CD47 expression was expressed on virtually all CLL cells and was similar in level of expression between CLL cells from phenotypically sensitive or resistant patient samples (**Figure 2A**). Total SIRPα expression was also similar between phenotypically sensitive and resistant NLCs (**Figure 2B**). Immuno-fluorescence analysis (**Figure 2C**) showed that virtually all NLCs expressed SIRPα (**Figure 2C**). Therefore, increases in the expression of CD47 or SIRPα does not account for the emergence of ADP-resistance. Overall, these data indicate that i) CD47 and SIRPα are expressed on leukemic CLL cells and NLCs respectively and ii) that differences in their expression profiles do not explain the ADP resistance phenotype.

### SIRP α Suppresses ADP in NLCs

**Figure 3:**
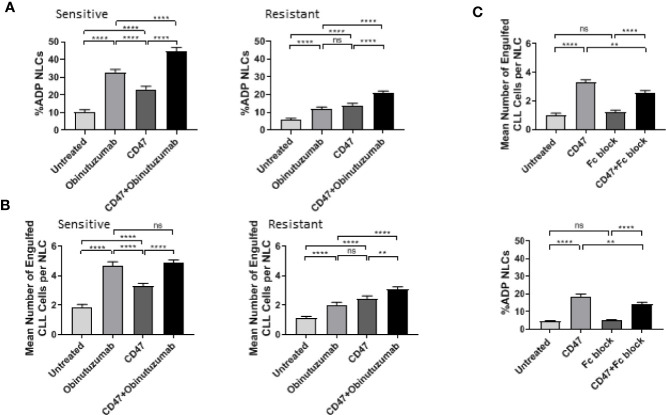
CD47 blockade enhances ADP response in NLCs. Phenotypically sensitive and resistant CLL PBMC cultures were treated anti-CD47 blockade (2.5 μg/ml) for 2 h before incubation with obinutuzumab (10 μg/ml) for an additional 2 h. Phagocytosis was quantitated as described in *Materials and Methods*. Data presented as the percent NLCs engulfing CLL cells as a fraction of the total pool of NLCs **(A)** or the number of engulfed CLL cells per phagocytosing NLC **(B)**. Data presented as mean value ± SEM from eight sensitive and nine resistant CLL patients. **(C)** CLL PBMCs were treated with Fc-block reagent and/or CD47 blocking antibody for 2 h before addition of obinutuzumab for an additional 2 h. ADP response was examined as outlined above. Data presented as mean value +/− SEM from six patients. Statistical analysis used a Mann Whitney U Test. **p < 0.01 and ****p < 0.0001. ns, not significant.

We examined whether a CD47 blocking antibody could derepress ADP responses in NLCs (**Figures 3A–C**). A 2 h pretreatment with CD47 blocking antibody (2.5 μg/ml) followed by addition of obinutuzumab (10 μg/ml) significantly increased the percentage of phenotypically sensitive NLCs that participated in ADP from 32 to 45% (P < 0.05; **Figure 3A**; **Supplementary Figure 3**). However, CD47 blocking antibody did not significantly enhance the number of phagocytic events/NLC beyond those seen with obinutuzumab alone in phenotypically sensitive NLCs (**Figure 3B**; **Supplementary Figure 3**). In contrast, a 2 h pretreatment with CD47 blocking antibody followed by obinutuzumab significantly increased the percentage of phagocytosing NLCs and significantly increased the number of phagocytic events/NLC in phenotypically resistant NLCs compared to obinutuzumab alone (P < 0.05; **Figure 3B**; **Supplementary Figure 3**). Thus, the phenotypically resistant NLCs appeared more responsive to CD47 blocking than were phenotypically sensitive NLCs. However, we noted that treatment with the murine IgG1 CD47 blocking antibody treatment alone (without obinutuzumab) significantly increased (P < 0.05) both the phagocytic events/NLC as well as the percent NLCs participating in ADP (**Figures 3A, B**; **Supplementary Figure 3**) compared to the untreated cohorts. This again, was more evident in the phenotypically resistant NLCs (**Figures 3A, B**; **Supplementary Figure 3**).

These results prompted us to examine whether the CD47 antibody was able to induce an FcγR-dependent ADP in NLCs derived from CLL patients. The CD47 induced phagocytosis by NLCs was significantly reduced by treatment with an Fc blocking antibody (**Figure 3C**, **Supplementary Figure 4**) indicating that the IgG1 CD47 antibody is able to induce to an FcγR-dependent ADP response. This latter observation indicated we could not use the CD47 blocking antibody to interrogate the SIRPα pathway. However, it should be noted that there was a significantly lower ADP response to CD47 blocking antibody alone than to obinutuzumab in phenotypically sensitive NLCs compared with resistant NLCs. This suggests CD47-mediated suppression of ADP may be more prominent in phenotypically resistant NLCs. Specifically, the CD47 Ab contributes to ADP responses both directly and indirectly. In the first instance the IgG1 component can directly activate ADP. This would be more noticeable in phenotypically sensitive NLCs than in phenotypically resistant NLCs. In the second instance, CD47 Ab can indirectly enhance ADP responses by derepressing the SIRPα-mediated suppression. This would be more noticeable in phenotypically resistant NLCs.

**Figure 4:**
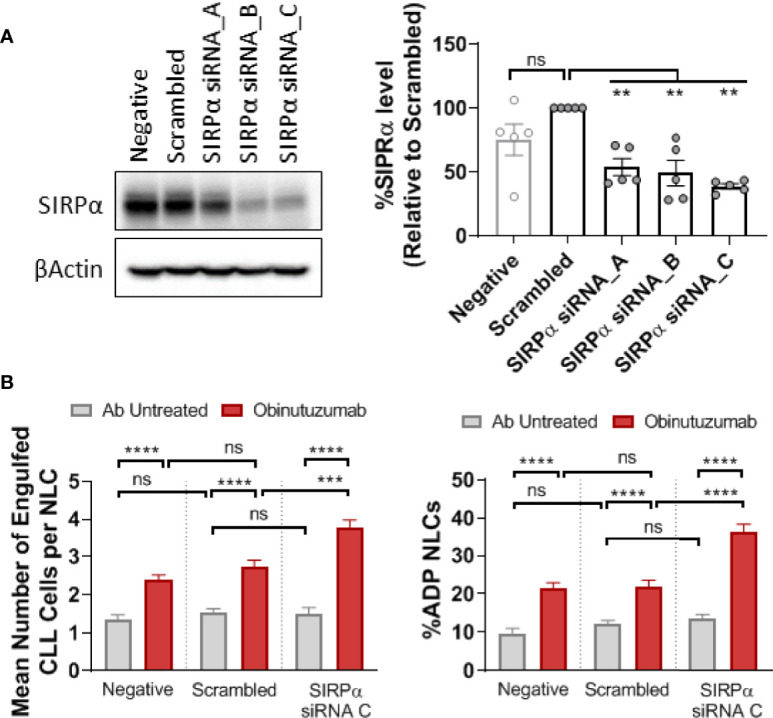
SIRPα knockdown enhances ADP response in NLCs. CLL derived NLCs were transfected on day 7 with 50 nM scrambled siRNA control or three different SIPRα selective siRNAs (siRNA A–C). **(A)** Protein was harvested 48 h post transfection and SIRPα and β-actin expression visualized and quantitated by western blot to examine knockdown efficiency. Data presented as mean value ± SEM and normalized to scrambled siRNA control. Each point represents an individual NLC donor. **(B)** NLCs were co-cultured with autologous CLL cells for 48 h after SIRPα siRNA C or scrambled siRNA transfection. Cultures were then treated with or without obinutuzumab (10 μg/ml) stimulation for 2 h before ADP responses were estimated. Data presented as the percent NLCs engulfing CLL cells as a fraction of the total pool of NLCs or the number of engulfed CLL cells per phagocytosing NLC. Data presented as mean value +/− SEM from nine patients. Statistical analysis used a Mann Whitney U Test. ***p < 0.001 and ****p < 0.0001. ns, not significant.

To determine whether the SIRPα/CD47 checkpoint is a negative regulator of ADP we transfected NLCs with siRNA targeting SIRPα. Initial screening of a suite of siRNAs identified a target sequence that reduced SIRPα protein expression by more than 60% compared to scrambled control (**Figure 4A**). We transfected phenotypically resistant NLCs with the targeting siRNA-C or a scrambled control and then treated the cultures with obinutuzumab (**Figure 4B**). The obinutuzumab-induced ADP response by NLCs is significantly enhanced (P < 0.5) following SIRPα knockdown compared to a scrambled siRNA control (**Figure 4B**; **Supplementary Figure 5**). Both the percent NLCs participating in ADP as well as the ADP events/NLC were increased following SIRPα knockdown (**Figure 4B**; **Supplementary Figure 5**). This established that SIRPα negatively regulates ADP responses in NLCs derived from CLL patients and suggests that SIRPα could potentially be targeted to reinstate sensitivity to obinutuzumab.

### SIRPα–Dependent Inhibition of ADP Response Is Mediated *via* Shp1

**Figure 5:**
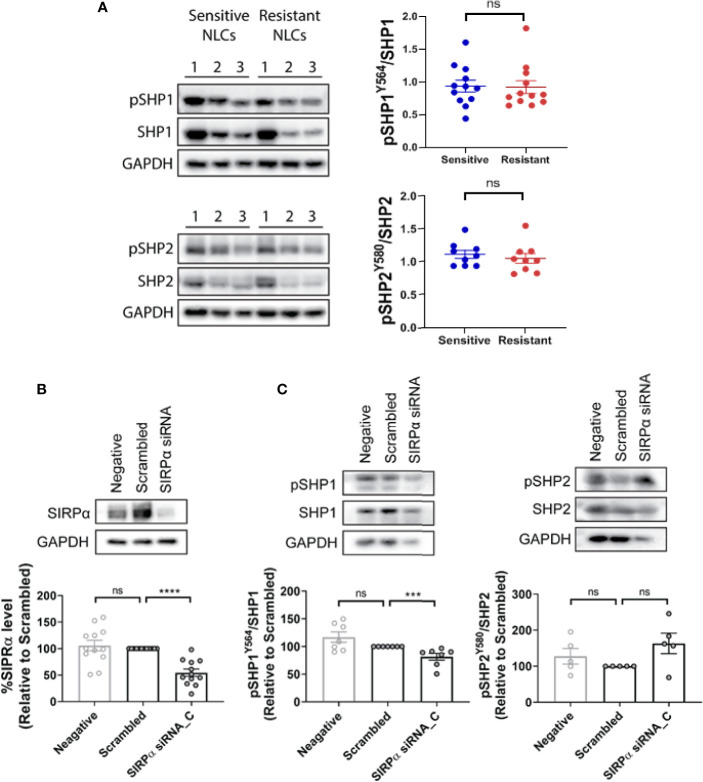
SHP1 but not SHP2 is downstream of SIRPα. **(A)** Total and phosphorylated SHP1 and SHP2 expression were examined in phenotypically sensitive and resistant NLCs by western blotting. Representative blots and quantified data are shown. Each point represents a single patient. **(B)** NLCs were transfected with SIRPα siRNA C or scrambled control siRNA. SIRPα protein expression, as well as **(C)** total and phosphorylated SHP1 (pSHP1) or SHP2 (pSHP2) expression was estimated in transfected NLCs. Representative blots are shown and quantified data shown graphically. Data presented as mean value ± SEM and normalized to scrambled siRNA control. Each point represents an individual patient. Statistical differences were calculated by a Mann Whitney U Test. ***p < 001 and ****p < 0.0001. ns, not significant.

**Figure 6:**
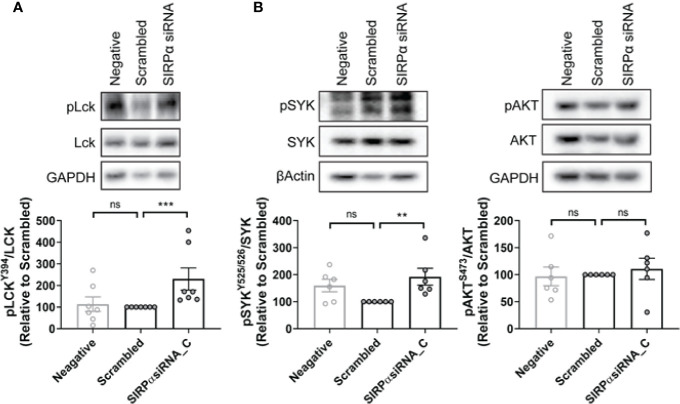
SYK but not AKT is associated with SIRPα-dependent repression of ADP in NLCs. **(A, B)** NLCs were transfected with 50 nM SIRPα specific siRNA C or scrambled control before the expression levels of phosphorylated Lck^Y394^, SYK, and AKT were examined by western blot. Representative blots are shown and quantified data shown graphically. Data presented as mean value ± SEM and has been normalized to scrambled siRNA control. Each point represents an individual patient. Statistical analysis used a Mann Whitney U Test. **p < 0.01 and ***p < 0.001. ns, not significant.

Shp1 and Shp2 are phosphatases implicated in multiple SH2-dependent signaling events, including SIRPα immune checkpoint signaling in macrophages (30–34). We examined the expression levels of Shp1, phospho-Shp1, Shp2, and phospho-Shp2 in NLCs by western blot and found them to be similar between phenotypically sensitive and resistant NLCs (**Figure 5A**; **Supplementary Figure 6**). Similarly, phospho-Shp1/Shp1 or phospho-Shp2/Shp2 ratios were similar between phenotypically sensitive and resistant NLCs (**Figure 5A**). Knockdown of SIRPα expression (**Figure 5B**) significantly reduced the phospho-Shp1/Shp1 ratio but not the phopsho-Shp2/Shp2 ratio compared to a scrambled siRNA control (**Figure 5C**). We next examined the effect of SIRPα depletion on phosphorylation of Lck at tyrosine 394, a Shp1-selective target (35, 36). SIRPα depletion significantly increased the level of phospho-Lck-Y394 (**Figure 6A**). Together, these data suggest that SIRPα modulates the Shp1 axis in NLCs, without engaging Shp2.

FcγR-dependent ADP occurs *via* two independent post-receptor signaling pathways (1, 2) i) SYK and BTK dependent (1) and ii) PI3K/p110δ/AKT dependent (2). To determine whether SIRPα-suppression engages these pathways we examined the effect of depletion of SIRPα on intracellular signaling *via* the SYK/BTK or PI3K/p110δ/AKT pathways. SIRPα depletion significantly enhanced activity of the SYK pathway as evidenced by the 1.8-fold increase in the phospho-SYK/SYK ratio compared with scrambled siRNA control (**Figure 6B**). In contrast, activity of the PI3K/p110δ/AKT pathway, as characterized by phospho-AKT/AKT level, was unchanged by SIRPα depletion compared to the scrambled siRNA control (**Figure 6B**). As these pathways have previously been shown to be independent post-receptor events, our results suggest that SIRPα suppresses ADP *via* a SYK dependent signaling pathway.

**Figure 7:**
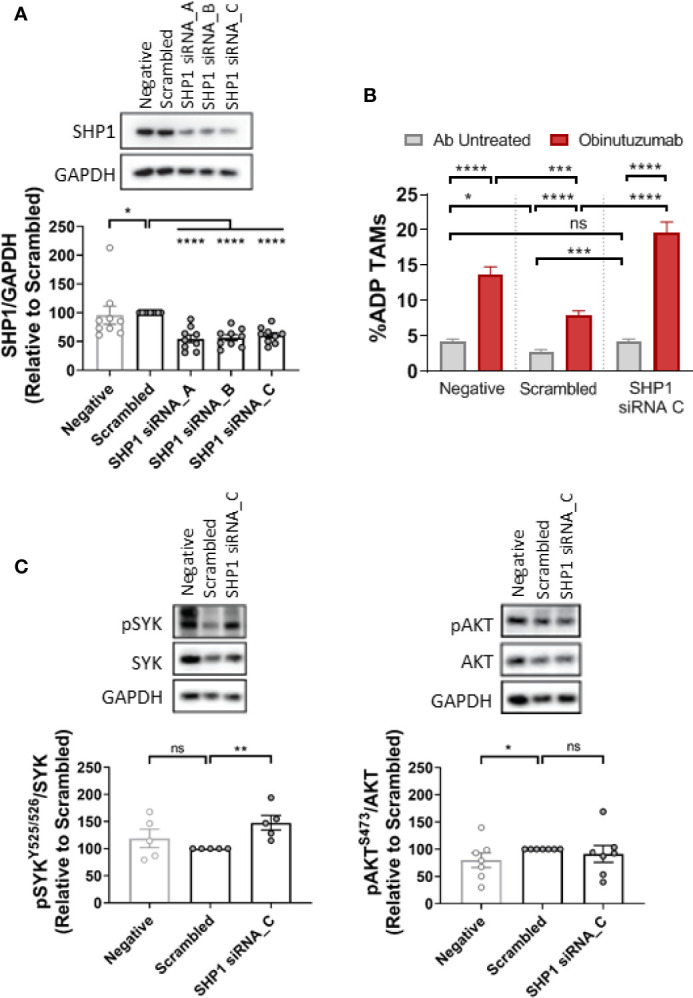
SHP1-dependent ADP repression is SYK-dependent. NLCs were transfected with 50 nM scrambled siRNA control or three different SHP1-siRNAs (siRNA A–C). **(A)** Protein was harvested after 48 h and SHP1 and GAPDH expression visualized and quantitated by western blot to examine knockdown efficiency. **(B)** NLCs were co-cultured with autologous CLL cells for 48 h after SHP1 siRNA C or scrambled siRNA transfection. Cultures were then treated with or without obinutuzumab (10 µg/ml) for 2 h before ADP responses examined. Data presented as mean value ± SEM from five CLL patients. **(C)** Western blot data analysis of SYK (pSYK^Y525/526^) and AKT phosphorylation (pAKT^S473^) in NLCs following SHP1 siRNA transfection. Representative blots and quantified data are shown. Data presented as mean value +/− SEM. Each point represents an individual patient. Statistical analysis used a Mann Whitney U Test. *p < 0.05, ***p, 0.001, and ****p < 0.0001.

Finally, to confirm the involvement of Shp1 in the SIRPα/SYK pathway we employed targeted depletion of Shp1 with siRNA (**Figure 7**). Initial screening of a suite of Shp1 siRNAs demonstrated we could significantly reduce Shp1 protein expression by 50% (**Figure 7A**) resulting in a significant increase of 50% in phospho-Shp1/Shp1 ratio (Supplementary **Figure 7**) compared to cells treated with a scrambled siRNA control. Shp1 siRNA treatment of NLCs significantly enhanced obinutuzumab-induced ADP compared with scrambled siRNA control (**Figure 7B**; **Supplementary Figure 8**). Moreover, Shp1 depletion significantly increased phospho-SYK/SYK^total^ 1.4-fold compared with scrambled siRNA control (**Figure 7C**). In contrast, Shp1 depletion did not alter phospho-AKT/AKT^total^ (**Figure 7C**). Finally, we incubated NLCs with the SYK-selective inhibitor, R406 (1) (**Supplementary Figure 9**). R406 reduced the phospho-SYK/SYK ratio but did not alter phopsho-Shp1/Shp1 (**Supplementary Figure 9**). Combined, these data demonstrate that Shp1 is a downstream effector of SIRPα and is an upstream negative regulator of SYK-mediated ADP activity in NLCs.

## Discussion

CD47 is a transmembrane protein which has recently been characteried as having anti-phagocytic properties which reduce tumor cell immunogenicity. The CD47:SIRPα immune checkpoint is thus a promising translational target for blood and solid tumours (37, 38). At present there are at least 13 phase 1 or 2 trials of CD47/SIRPα targeting agents (18). Early clinical trials with CD47 blocking antibodies have shown therapeutic promise but have also been associated with off target toxicities (23, 27). To circumvent the off-target effects of CD47 blocking agents there has been increasing interest in the CD47 receptor, SIRPα (25). SIRPα, is selectively expressed on myeloid cells (including macrophages such as NLCs). Thus, identifying the effectors of SIRPα-dependent repression of ADP responses may identify actionable intracellular targets that antagonize the SIRPα axis. In this study we show that i) the SIRPα checkpoint suppresses ADP responses to obinutuzumab by NLCs from CLL patients, ii) SIRPα signaling operates *via* a Shp1/SYK axis, and, iii) SIRPα-mediated suppression does not affect PI3K/p110δ/AKT–dependent ADP.

NLCs are specialized macrophages derived from circulating monocytes from CLL patients (39–41). Using NLCs from CLL patients we show that SIRPα-driven suppression is mediated *via* downstream activation of Shp1 which in turn dephosphorylates SYK to dampen the ADP response. The most obvious proof of this is reflected by the enhancement of ADP responses and hyper-phosphorylation of SYK following SIRPα or Shp1 knockdown in NLCs. In addition, inhibition of SYK results in suppressed ADP responses but did not alter Shp1 phosphorylation indicating Shp1 is upstream of SYK. This is consistent with a previous study showing that Shp1 was a negative regulator of SYK in macrophage cell lines (42). The Shp1 selectivity, identified in this study, may represent a point of difference between NLCs and other myeloid cell systems. For instance, SIRPα has been shown to signal through both Shp1 and Shp2 (18, 43, 44). In our study, Shp2 phosphorylation was unaltered by SIRPα depletion and SIRPα depletion induced phosphorylation of the Shp1-selective target Lck^Y394^ suggesting that SIRPα-dependent ADP suppression, in NLCs, is predominantly, if not exclusively, Shp1-dependent. Thus, exploiting the Shp1-dependence of SIRPα signaling in NLCs may add an extra level of selectivity when targeting the defective ADP response in CLL patients.

**Figure 8:**
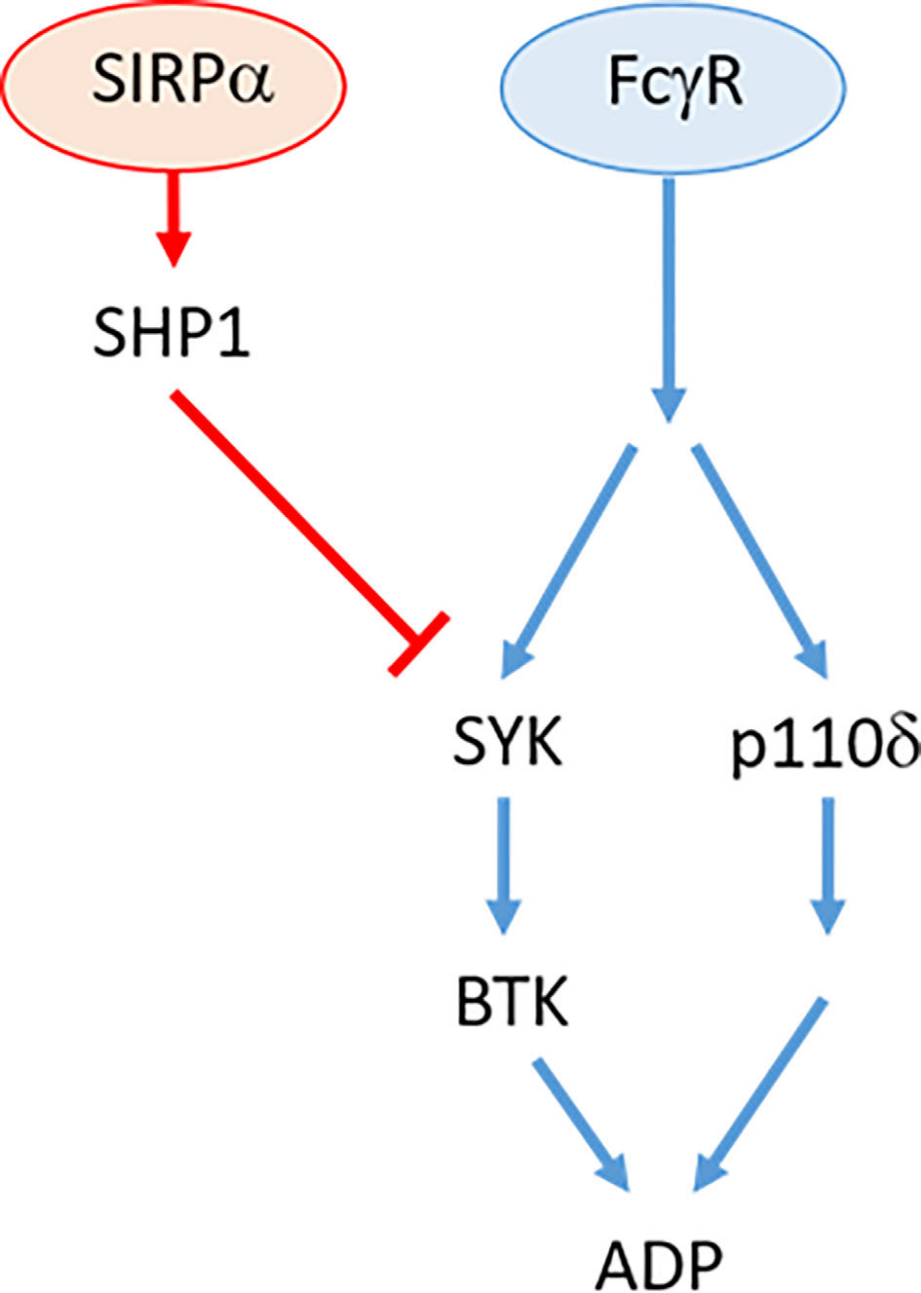
Overview of FcγR signaling pathway in antibody resistant NLC. Therapeutic antibody binding to cell-surface receptor, FcγR independently activates SYK/BTK and p110δ/AKT pathways. Activation of SIRPα by tumor cell CD47 (not shown) activates SHP1 phosphatase that suppresses SYK activity.

FcγR-dependent ADP responses to therapeutic antibodies have been shown to be mediated *via* two independent signaling pathways (1, 2) (**Figure 8**). One pathway is mediated *via* SYK/BTK which becomes increasingly repressed during disease progression (1). The SYK/BTK pathway is subject to multiple suppressive effectors such as Ship1, HDAC7, SIRPα, or Shp1 (1, 17) (this study; **Figure 8**). The second pathway is mediated *via* PI3K/p110δ/AKT (2) and does not appear to be subject to any known negative regulators in NLCs (2). This has implications for our understanding of the molecular events regulating FcγR-dependent ADP. Specifically, the divergence of the two pathways indicates that separate signaling and suppressive events are instigated in the immediate post-receptor environment. In this regard, little is known about the immediate post-receptor events that transduce the FcγR signal in macrophages. It is known that FcγR ligation induces phosphorylation of SH2 domains within the ITAM regions resulting in recognition by adaptor proteins and subsequent signal transduction (45, 46). Of potential relevance here is the recent report by Chen *et al.* showing that SIRPα-dependent suppression of phagocytosis acts *via* a negative acting interaction of SLAMF7 with Mac1 (24). How this relates to the pathways described in this study remains to be elucidated but does identify a potential effector that may operate in the immediate post-receptor environment in NLCs (24).

It is interesting to note that dampening of the ADP response, that occurs during CLL disease progression, is attributable exclusively to selective disruption of the negative regulatory pathways (e.g. SIRPα, HDAC7, ship1) rather than the activating pathways (e.g. SYK, BTK, PI3K, SLAMF7). The etiological basis for this imbalance is unclear but not unprecedented since a similar situation exists for the “classical” immune checkpoint of T cells in which the suppressive arm becomes prominent in cancers (47). Whilst classical blocking strategies such as antibodies and soluble ligands have been the main focus of clinical trials our study and others highlight the potential value of small molecular weight targeted agents. For example, RRx-001 is a small molecular weight drug showing promise in recent clinical trials in various cancer types (48). RRx-001 switches the macrophage phenotype from M2 to M1 and causes a decrease in expression of SIRPα and enhanced tumor clearance by macrophage phagocytosis *in vivo* (48). This provides strong evidence that the CD47:SIRPα axis can be targeted using small molecular weight drugs. Another strategy has reported the efficacy of small molecular weight agents targeting the synthesis and trafficking of CD47 in cancer cells (49). We now report an additional strategy to combine traditional antibody therapies with agents targeting suppressors of the innate immune checkpoint such as SIRPα, and Shp1 or the previously identified suppressors such as, Ship1 (1) or HDAC7 (17). Such strategies have the advantage of being selective for myeloid cells, in which the innate immune checkpoint is suppressed, and avoids the expense incurred with antibody therapies.

In contrast to the potential intracellular targets, identified in our study, strategies targeting the extracellular components of the CD47:SIRPα immune checkpoint are already in clinical trial for blood and solid tumors. The clinical development and early trials were driven by encouraging results in preclinical models of breast cancer, melanoma, lung cancer, and multiple blood cancer types (18). At least seven phase 1/2 trials of CD47 (e.g. Hu5F9-G4 or TTI-662)/SIRPα (e.g. CC-95251) targeting agents, as monotherapies, and six Phase 1/2 trials of combination therapies are underway (18). The results of our study suggest that combinations of traditional antibody therapies, such as rituximab or obinutuzumab, plus a CD47:SIRPα blocking agent may be efficacious in CLL. In this regard there are several early phase clinical trials currently recruiting for combinations of CD47:SIRPα blockers + therapeutic antibodies for solid tumors and blood cancers (Feng et al., 2019). Moreover, a recent phase 1b clinical trial combining rituximab with an anti-CD47 therapeutic antibody (Hu5F9-G4) showed promising results in lymphoma patients (27). Overall, these early phase safety/toxicity studies have shown that blocking the innate immune checkpoint is a promising new therapeutic strategy for blood cancers (18).

In conclusion, we have identified the SIRPα:Shp1 axis as a selective actionable target that could be exploited using intracellularly and extracellularly directed strategies to enhance ADP responses, by NLCs, to existing therapeutic antibodies in CLL patients.

## Data Availability Statement

The raw data supporting the conclusions of this article will be made available by the authors, without undue reservation.

## Ethics Statement

The studies involving human participants were reviewed and approved by metro south Health Human Research Ethics Committee. The patients/participants provided their written informed consent to participate in this study.

## Author Contributions

Y-CC: performed experiments, contributed to design of studies, contributed to data analysis and interpretation, contributed to writing manuscript. MB: performed experiments and provided technical advice, contributed to data analysis and interpretation. SM: coordinated sample collection, contributed to study design, contributed to data analysis and interpretation, contributed to writing manuscript. PM: Coordinated sample collection, contributed to study design, contributed to data analysis and interpretation, contributed to writing manuscript. DG: coordinated sample collection, contributed to study design. AB: provided technical advice and guidance as well as co-writing the manuscript, contributed to data analysis and interpretation. NS: directed design of the study, contributed to data analysis and interpretation, contributed to manuscript writing. All authors contributed to the article and approved the submitted version.

## Conflict of Interest

The authors declare that the research was conducted in the absence of any commercial or financial relationships that could be construed as a potential conflict of interest.
