# Supplementary material for: SIRPα Suppresses Response to Therapeutic Antibodies by Nurse Like Cells From Chronic Lymphocytic Leukemia Patients
Source: Front Immunol. 2021 Jan 21;11:610523. doi: 10.3389/fimmu.2020.610523 (PMC7859087; doi:10.3389/fimmu.2020.610523)
Supplement: Supplementary file 1 [file DataSheet_1.pdf]

Supplementary Figures 1 – 8

Chen YCE et al

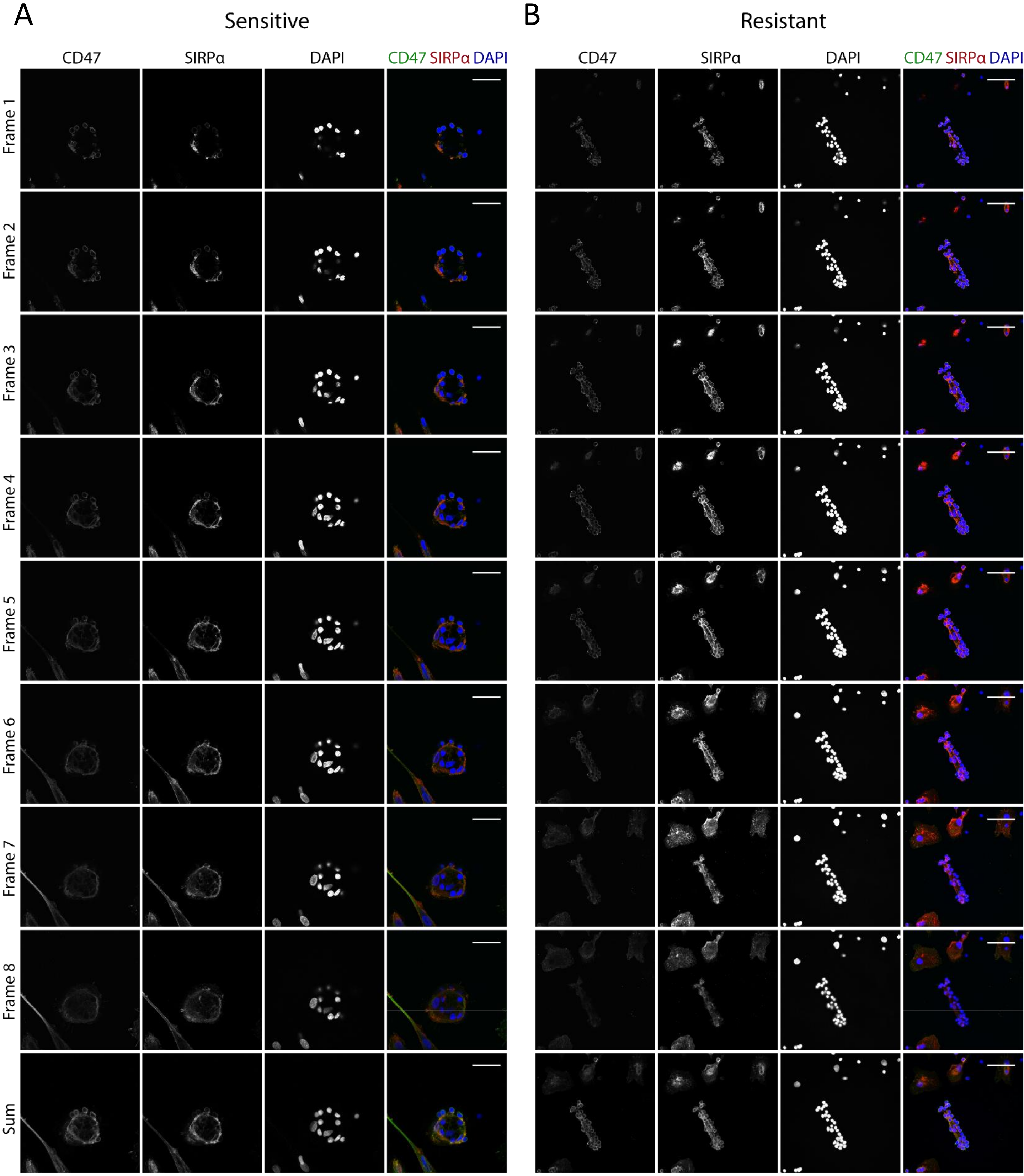

Supplementary Figure 1. Z-stack of obinutuzumab treated CLL PBMC cultures. CLL PBMCs were cultured for 7 days before incubation with obinutuzumab (10µg/ml) for 30 minutes. Non-adherent cells were removed before immunofluorescent staining of antibody sensitive (Supplementary Figure 1A) and antibody resistant (Supplementary Figure 1B) CLL PBMC cultures. Z-stacking was performed using confocal microscopy. Images were taken on several planes at 0.84um slices. Cells staining positive for SIRPα appear red, CD47 appear green and nuclear staining appears blue (DAPI). Each frame and the sum of all frames is shown; x100 magnification. Scale bar = 100µm.

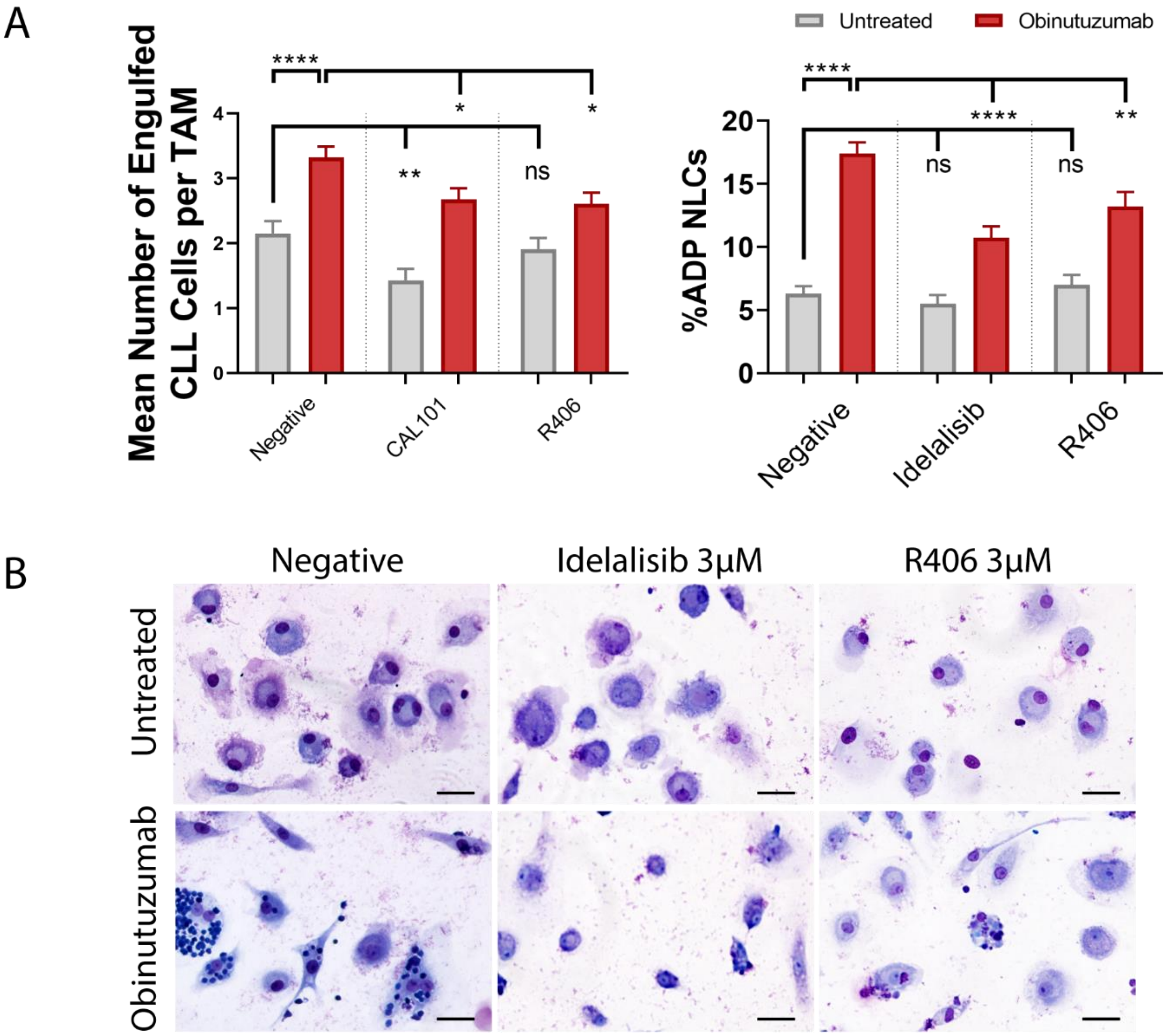

Supplementary Figure 2. SYK and PI3K/p110δ are mediators of FcγR pathway in NLCs. Phenotypically sensitive or resistant CLL PBMC cultures were treated with SYK inhibitor (R406) or PI3K/p110δ inhibitor (Idelalisib), both at 3μM for 2 hr before 2hr incubation with obinutuzumab (10μg/ml). NLCs were stained with May-Grunwald-Giemsa stain and phagocytosis was quantitated as described in "materials and methods". (**A**) Quantified data presented as the percent NLCs engulfing CLL cells as a fraction of the total pool of NLCs or the number of engulfed CLL cells per phagocytosing NLC. Data presented as mean value ± SEM from six patients in each group. (**B**) Representative images of an individual patient sample is shown. Statistical analysis used an unpaired Mann-Whitney U test **p < 0.01 and ****p<0.0001. Scale bar = 25μm.

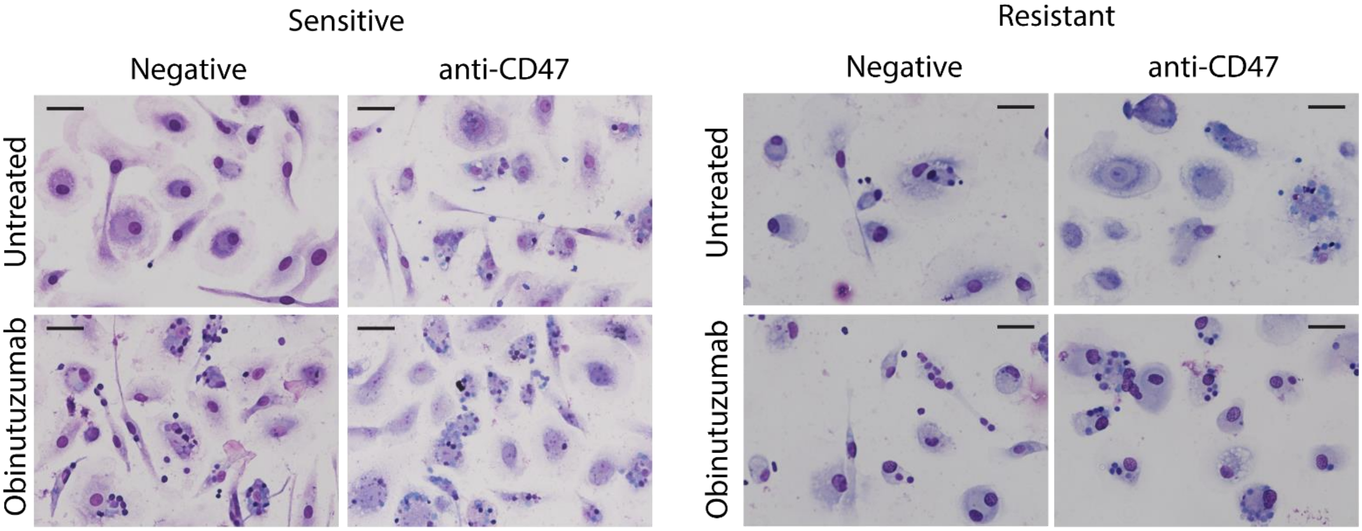

Supplementary Figure 3. CD47 blockade enhances ADP response of NLCs. Phenotypically sensitive and resistant CLL PBMC cultures were treated with anti-CD47 blockade (2.5µg/ml) for 2 hours on day 7 before incubation with or obinutuzumab (10µg/ml) for an additional 2hours. NLCs were stained with May-Grunwald-Giemsa. Representative images from a single sensitive and resistant patient are shown. Scale bar = 25µm.

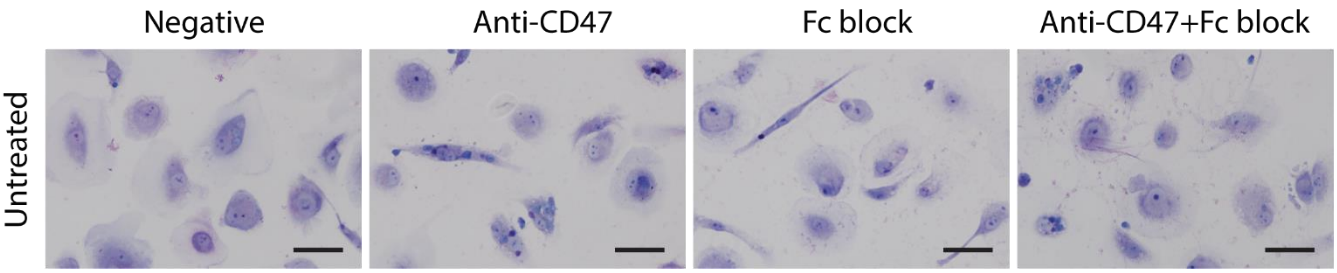

Supplementary Figure 4. CD47 blockade enhanced ADP response in NLCs is an FcγR dependent event. CLL PBMC were treated with anti-CD47 blockade and/or Fc blockade for 2 hours and incubated with obinutuzumab (10μg/ml) for an additional 2 hours. CLL cells were removed prior to NLCs were stained with May-Grunwald-Giemsa stain. Phagocytosis was examined as described previously. Scale bar = 25μm.

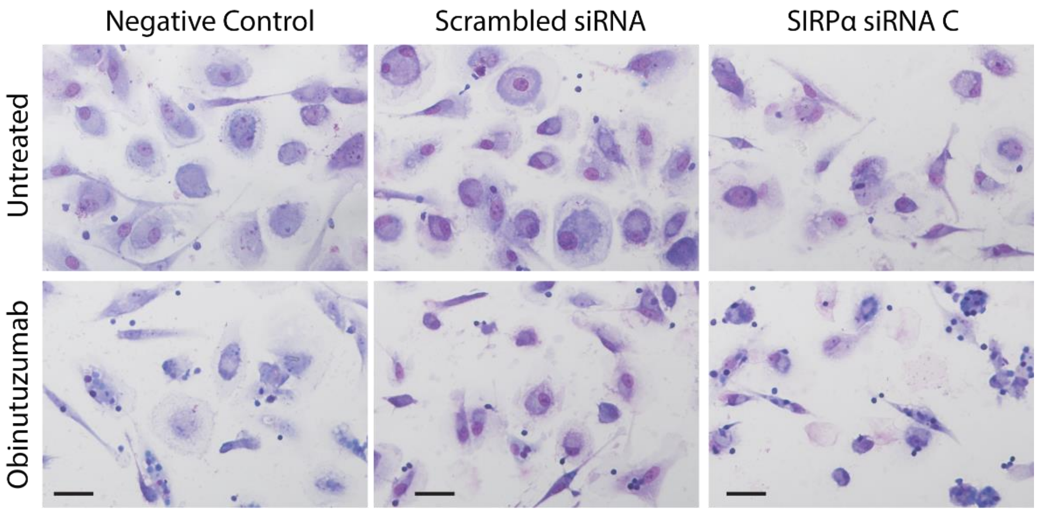

Supplementary Figure 5. SIRPα knockdown enhances ADP response in NLCs. NLCs were co-cultured with autologous CLL cells for 48 hours after scrambled siRNA control or SIRPα siRNA C transfection. Cultures were then treated with or without obinutuzumab (10µg/ml) for 2 hours before May-Grunwald-Giemsa staining. Representative images from a single patient is shown. Scale bar = 25µm.

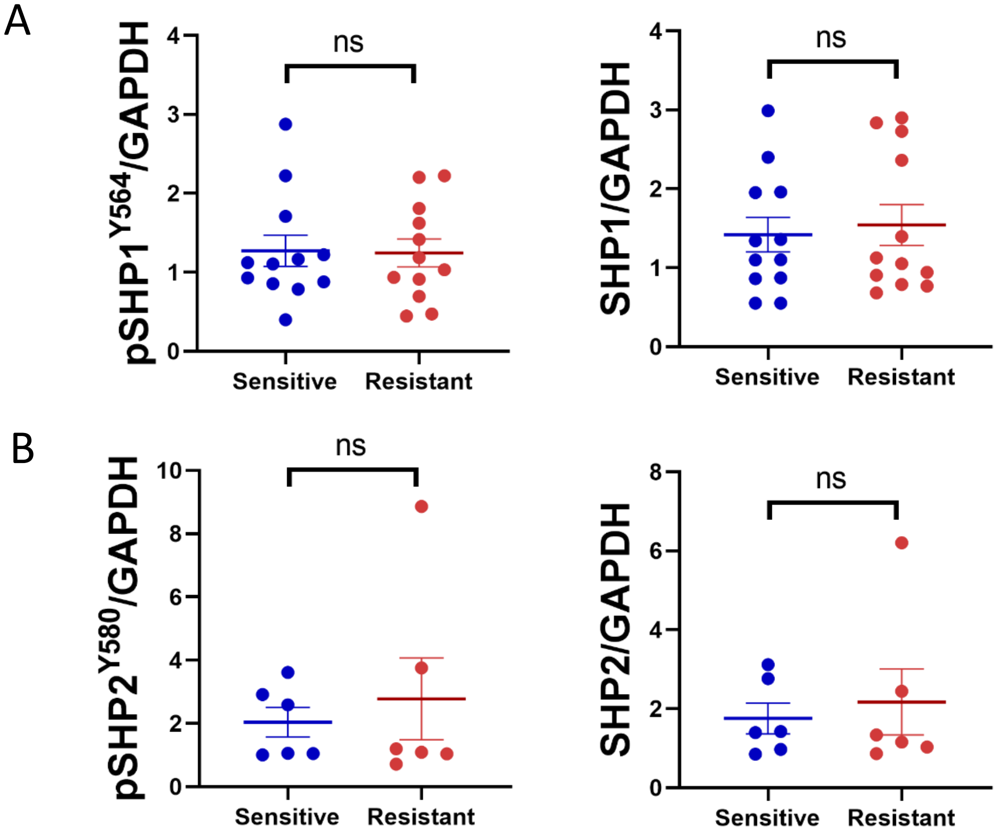

Supplementary Figure 6. SHP1 and SHP2 expression level are similar in sensitive and resistant NLCs. Phenotypically sensitive and resistant NLCs were harvested on day 7 for western blotting. **(A)** Normalisation of total and phosphorylated SHP1 by GAPDH were examined in phenotypically sensitive and resistant NLCs. **(B)** Total and phosphorylated SHP2 expression was examined. Data presented as mean value ± SEM and normalized to GAPDH. Each point represents an individual patient. Statistical analysis used an unpaired Mann-Whitney U test *$p < 0.05$.

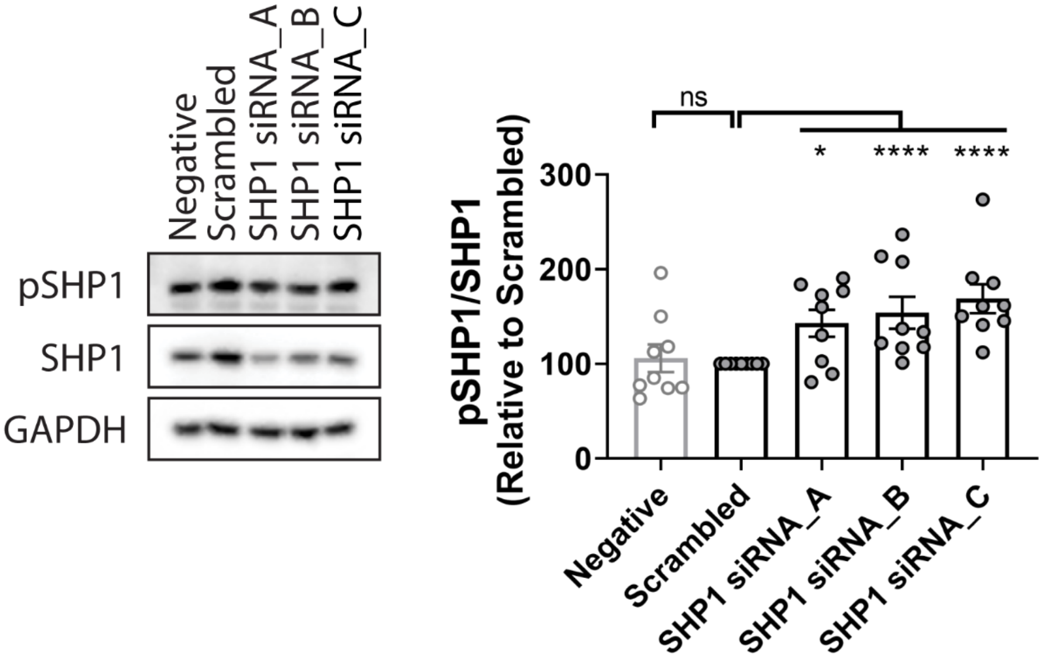

Supplementary Figure 7. SHP1 knockdown increases SHP1 phosphorylation activity. NLCs were transfected with scrambled siRNA control or three different SHP1-siRNAs (siRNA A-C). NLCs were collected after 48 hours for analysis of SHP1 (Tyr564) phosphorylation by western blot. Representative blots are shown and quantified data is shown graphically. Data presented as mean value ± SEM. Each point represents an individual patient. Statistical analysis used an unpaired Mann-Whitney U test . *p < 0.05, ****p<0.0001.

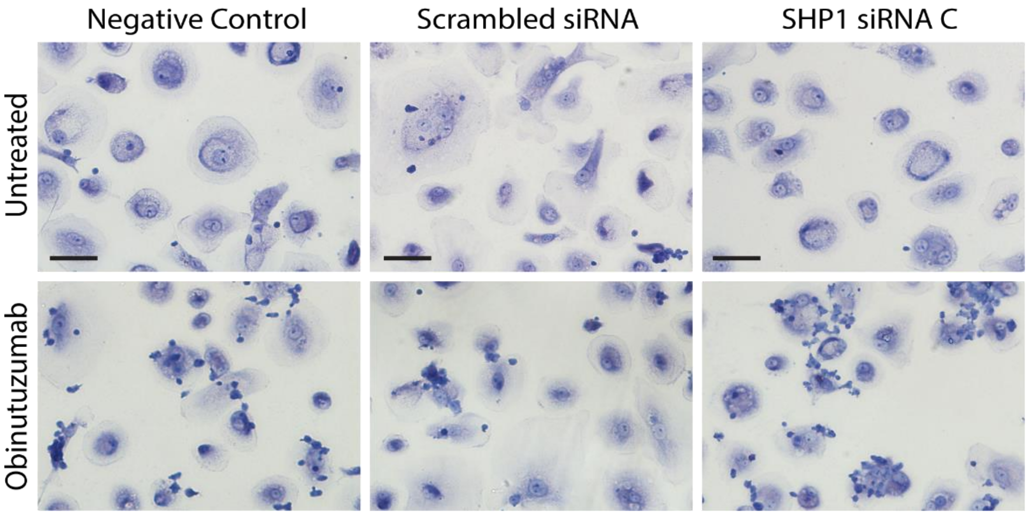

Supplementary Figure 8. SHP1 knockdown enhances ADP response in NLCs. NLCs were co-cultured with autologous CLL cells for 48 hours after SHP1 siRNA C or scrambled siRNA transfection. Cultures were then treated with or without obinutuzumab (10µg/ml) for 2 hours before May-Grunwald-Giemsa staining. Representative images from a single patient is shown. Scale bar = 25µm.

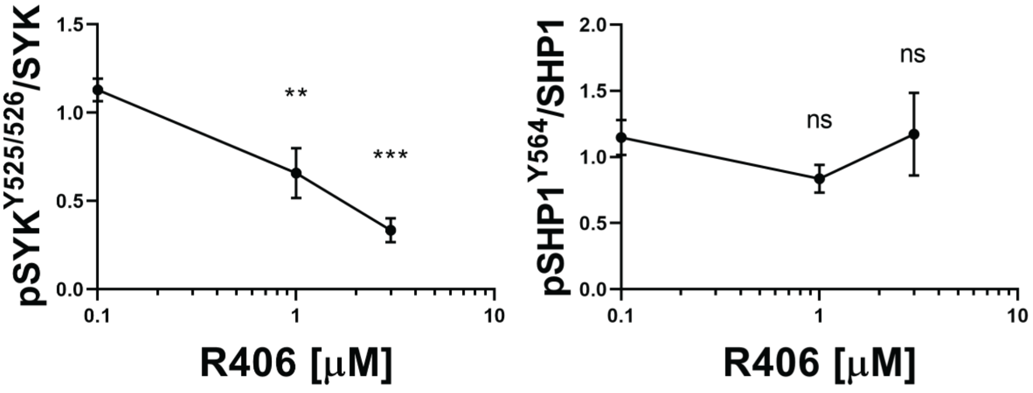

Supplementary Figure 9. SYK is downstream of SHP1. NLCs were treated with differing doses of the SYK-selective inhibitor, R406, for 2 hours. NLCs were harvested and the relative level of pSYK$^{Y525/526}$/SYK or pSHP1^{Y564}/SHP1 was estimated from the western blot data. Data presented as mean value ± SEM from 3 patient samples. Statistical analysis used an one-way ANOVA followed by Dunnett's test $^{**}p < 0.01$ and $^{***}p<0.001$.
